# How Are Wearable Activity Trackers Adopted in Older Adults? Comparison between Subjective Adoption Attitudes and Physical Activity Performance

**DOI:** 10.3390/ijerph17103461

**Published:** 2020-05-15

**Authors:** Byung Cheol Lee, Junfei Xie, Toyin Ajisafe, Sung-Hee Kim

**Affiliations:** 1 Department of Engineering, Texas A&M University-Corpus Christi, Corpus Christi, TX 78412, USA; byungcheol.lee@tamucc.edu; 2 Department of Electrical and Computer Engineering, San Diego State University, San Diego, CA 92182, USA; jxie4@sdsu.edu; 3 Department of Kinesiology, Texas A&M University-Corpus Christi, Corpus Christi, TX 78412, USA; toyin.ajisafe@tamucc.edu; 4 Industrial ICT Engineering, Dong-eui University, Busan 47340, Korea

**Keywords:** activity tracker, adoption attitude, step count, MVPA time, long-term use

## Abstract

Wearable activity trackers can motivate older adults to engage in the recommended daily amount of physical activity (PA). However, individuals may not maintain their use of the trackers over a longer period. To investigate the attitudes of activity tracker adoption and their effects on actual PA performance, we conducted a three-month study. We gave activity trackers to 16 older adults and assessed attitudes on activity tracker adoption through a survey during the study period. We extracted participants’ PA measures, step counts, and moderate and vigorous physical activity (MVPA) times. We observed significant differences in adoption attitudes during the three different periods (*χ*^2^(2, 48) = 6.27, *p* < 0.05), and PA measures followed similar decreasing patterns (*F*(83, 1357) = 12.56, 13.94, *p* < 0.00001). However, the Pearson correlation analysis (*r* = 0.268, *p* = 0.284) and a Bland–Altman plot indicated a bias between two PA measures. Positive attitudes at the initial stage did not persist through the study period, and both step counts and length of MVPA time showed waning patterns in the study period. The longitudinal results from both measures demonstrated the patterns of old adults’ long-term use and adoption. Considering the accuracy of the activity tracker and older adults’ athletic ability, MVPA times are more likely to be a reliable measure of older adults’ long-term use and successful adoption of activity trackers than step counts. The results support the development of better activity tracker design guidelines that would facilitate long-term adoption among older adults.

## 1. Introduction

Regular exercise and increased physical activity (PA) are crucial in maintaining physical and mental wellbeing and in lowering the risk of obesity in older adults. Generally, regular PA enhances muscular and cardiorespiratory fitness and lowers the risk of depression and the decline of cognitive functions [1,2]. However, low levels of PA have been associated with chronic disease, such as cardiovascular disease, diabetes, and certain cancers [3,4,5]. Approximately 35% of older adults in the United States aged 65 and over between 2007–2010 are obese [6], and almost two thirds of older adults do not recognize the importance of daily PA [4,7]. Less than 15% of older adults satisfied national guideline recommendations, which include at least 150 min of moderate-intensity aerobic activity, or 75 min of vigorous-intensity aerobic activity and two or more days of muscle strengthening activities per week [8,9]. A national survey of self-reported data illustrated that almost 84% of adults over 65 in the United States are physically inactive [10]. To mitigate these challenges, we need to develop better and more sustainable interventions and policies that encourage more regular exercise for older adults.

The step count and the amount of energy expenditure performing daily activities are associated with frailty levels [11]. Frailty is defined as a clinical condition wherein individual functional resources in physical, psychological or social domains is depleted [12]. Frailty makes older adults more vulnerable and susceptible to adverse health outcomes and mortality [13]. Physical frailty may increase the risk of physical disability, poor quality of life and even death [5]. Indeed, frail older adults spend more time in a condition of a sedentary lifestyle in comparison with healthy older adults [14]. In addition, previous longitudinal studies have found that older adults with lower physical activity levels have a higher risk of mortality than those with higher physical activity levels [15,16]. Yamamoto et al. [17] suggested that a high daily step count is associated with a lower risk of all-cause mortality in physically independent Japanese elderly people. They suggested that higher physical activity levels have preventive effects on all-cause mortality. Therefore, daily step counts can be a valid assessment of the level of physical activity in older adults’ daily lives.

As a viable solution to promote PA in older adults, an activity tracker is suggested. The activity tracker is a relatively affordable pedometer that automatically records step counts and can provide instant feedback to users. Increasing the daily step counts can be a practicable approach to increase overall PA levels and reducing sedentary behavior [18,19]. Thanks to advanced sensor technologies and data analysis techniques, various behavioral change techniques such as goal setting, social networking, feedback, and rewards are embedded in the activity tracker [20,21,22]. It can also track longitudinal PA levels for each individual. This can enable healthcare professionals to tailor PA recommendations, thereby encouraging the maintenance of PA behavior.

Despite the advantages of an activity tracker, long-term adoption rates are still low. Herrmann and Kim reported that over 50% of activity tracker users stopped wearing them within six months, and less than 20% kept using them after six months [23]. Ledger and McCaffrey found that more than half of the users who purchased activity trackers abandoned them within the first two weeks, and approximately two thirds stopped tracking their activity records within six months [24]. These poor adoption patterns mirror those of older adults. The American Association of Retired Persons (AARP) reported that 75% out of 92 participants stopped using their activity trackers after a month, and only half indicated their intention to adopt activity trackers on a long-term basis [25]. Fox and Duggan [26] found that less than 2% of respondents older than 65 have taken advantage of current activity logging or monitoring technology. Overall, one in three activity tracker users of all adult ages stop using the device within six months after purchase [24,27]. 

Prior research has suggested that discontinuing use of activity trackers is caused by complex and multidimensional aspects. Ridgers et al. [28] concluded that activity trackers are generally evaluated positively in terms of effectiveness and feasibility, but more longitudinal research is needed to verify actual long-term adoption. Researchers have investigated the adoption of commercial activity trackers in older adults and discussed the adoption barriers mainly through qualitative measures such as experience, usefulness, ease-of-use, and attractiveness [29,30,31]. Instead of simple design specifications or functional improvements to the device, researchers should examine the lack of interest in gathering exercise data, additional burdens while using trackers, and age-specific demands [32]. In addition to identifying the multidimensional factors leading to initial usage, less is known about how the adoption of an activity tracker directly affects PA levels [33,34]. A thorough understanding of this relationship between attitudes and actual use could increase the researcher’s ability to predict device use and facilitate long-term activity tracker use. 

The goal of this study was to explore the adoption of activity trackers through comprehensive approaches: subjective measures by attitude surveys and performance measures by step counts and moderate and vigorous physical activity (MVPA) time analysis. In this study, we analyzed the following: (a) 16 older adults’ longitudinal attitude patterns toward adopting the wearable activity tracker; and (b) the effects of the activity tracker use in the degree of PA level variation (step counts and MVPA time) over a three-month period. The results provide insight into acceptance over time of the activity tracker as well as perceived advantages and disadvantages in older adults. The results could guide effective deployment strategies to promote long-term use and maximize benefits. To date, no study has attempted to connect the variability of PA with long-term adoption attitudes and usage intentions. Examining attitudes and intentions about activity trackers over time and actual usage pattern analysis may be particularly valuable for uncovering both barriers to and facilitators of older adults’ tracker adoption.

## 2. Materials and Methods 

### 2.1. Participants and Anthropometric Data

Participants of the current study were recruited from a local community fitness center in South Texas. Twenty older adults (14 females and 6 males) aged 65 to 77, were initially recruited but 16 participated. The inclusion criteria were that participants were over 65 years old, had no history of neurological disorders or other disabilities that could affect the subject’s movements, and were in generally good health. Participants also needed to have a regular exercise regimen and no previous experience of using activity trackers. 

Prior to participation, volunteers received a verbal and written explanation of the study’s protocol and written consent was obtained. To determine whether volunteers were eligible to participate in this study, the Paffenbarger Physical Activity Questionnaire (PPAQ) [35] and General Health History Questionnaire [36] were administered. Their body weight (kg) and height (m) were measured in light clothing (e.g., shorts and t-shirt) and without shoes. From the measured weight and height, their body mass index (BMI) was calculated. The Texas A&M University–Corpus Christi Institutional Review Board approved the research protocol (44–19).

### 2.2. Activity Tracker Adoption Survey Instrument

We assessed the attitudes on activity tracker adoption three times, and we administered a survey questionnaire containing 36 questions that were measured on a 7-point Likert scale at the beginning (i.e., week 1), middle (i.e., week 7), and end (i.e., week 13) of the study. The survey was adapted from the survey scale developed for the individual adoption of healthcare wearable devices [37]. The original survey scale was empirically developed to assess the factors of individuals’ adoption intentions toward healthcare wearable devices with the perspective of the privacy calculus model. The instrument assesses adoption attitudes and intentions using risk-benefit analysis between perceived privacy risk and perceived benefits. We modified some terms to make the survey more pertinent to the activity tracker adoption context.

The survey conducted psychometric tests by adopting the structural equation modeling (SEM) method to analyze the empirical data collected from 333 participants [37]. As main constructs, health information sensitivity, personal innovativeness in IT, legislative protection, perceived prestige, and perceived informativeness on individuals’ perceived privacy risk were identified. The impacts of perceived informativeness and functional congruence on individuals’ perceived benefits were also estimated. The survey suggested guidelines on improving policies to promote the adoption of wearable devices in the healthcare sector, delivering more effective healthcare services.

The reliability and validity of the survey items were evaluated by confirmatory factor analysis. Cronbach’s α scores for constructs ranged from 0.841 to 0.866, composite reliability value of each construct was higher than the threshold 0.6, and average variance extracted (AVE) of all constructs exceeded the recommended value 0.5. These metric values indicate sufficient reliability of the measurement model. In addition, validity was measured by content validity and construct validity. Since the variables in the survey were all derived from existing research, they exhibited good content validity. Convergent validity and discriminant validity were assessed by the item loading coefficient and the square root of AVE values, and the results indicated sufficient construct validity. 

### 2.3. Activity Tracker Used in This Study

For the activity tracker, we chose Withings Go, a wrist-worn device that can measure sleep patterns, steps taken, calories burned, elevation climbed, and distance travelled. The tracker can monitor and measure various activities (e.g., walking, running, swimming) and can record sleep quality and duration. It also provides distance data and calories burned using a manufacturer algorithm deployed via an ad hoc application programming interface (API). 

The adoption and usability of the activity tracker can be heavily dependent on the specific model and/or brand of the activity tracker. We selected the Withings Go for this study due to its greater-than-6-month battery life and user-friendly interface. This precludes the need for charging or replacing the battery during the study period and only requires minimal controls to function. It also provides visualized data in a simple format to make reading and interpreting activity patterns easier for older adults. Furthermore, users can conveniently set activity goals using a mobile app.

### 2.4. Experiment Procedure

After signing an informed consent agreement, the participants received an activity tracker with instructions developed by the research team on how to set up their daily step-count goal and how to use the tracker. The participants were asked to wear their trackers on their nondominant wrists for three months. They were instructed to avoid modification to their regular routine and frequently assess their comfort level. 

During the following 13 weeks, the research team met with the participants every two weeks and transferred their PA data to the manufacturer’s cloud storage. The research team provided support for device setup, including creating user accounts and familiarizing participants with basic functions and features.

### 2.5. Data Analysis

The responses from the activity tracker adoption survey were reported in means (M) and standard deviations (SD). Because of the limited number of participants and the lack of a normality condition, we conducted a Friedman test to assess the mean differences in ratings at the three evaluation periods. The Friedman test controls experimental variability among subjects, thus increasing the power of the test. Increased (or decreased) measurements in a subject provide the temporal variations of the adoption attitude in the overall study period. Dunn’s test was administered as a post-hoc test, which compares the actual difference in the sum of ranks with the expected average difference.

We accessed step count and activity duration data using the manufacturer’s API. For each participant during the study period, we obtained the number of steps achieved each day and the specific amount of time between the designated start and end times of activities as direct measures of tracker usage, and we used these data to estimate the mean daily step counts and activity durations. We assumed that participants had detached the tracker or did not exercise at all if their step counts were fewer than 500, which is less than the average daily step count for women over 85 years old [38]. Therefore, we excluded daily step counts of fewer than 500 from the analysis. 

The MVPA time is defined as the time that a participant walks or runs more steps than the minimum MVPA step counts. We assumed that fewer step counts than the minimum step count were inactive or sedentary periods. The minimum MVPA step count was computed using the method by McMahon et al. [39]. MVPA times were measured by login data, and step counts were collected by the numbers recorded in the tracker API. In the analysis, we converted the participants’ daily step count and average MVPA time to standardized variables to minimize the effects of individual differences. We calculated the standardized step count rate and standardized MVPA time rate per participant using the following formulae:

Standardized step count rate = (daily step count − average step count per participant)/average step count per participant(1)

Standardized MVPA time rate = (daily MVPA time − average MVPA time per participant)/average MVPA time per participant(2)

We assessed variability in daily step counts and MVPA time in three ways. First, descriptive statistics (mean and standard deviation) of step counts and MVPA times by each participant were given to determine PA levels using the weekly exercise routines of the participants. Second, we used a one-way repeated ANOVA to compare the variance in standardized daily step count to the variance in MVPA time rates and examine the nature of the association between the two measures. Third, we constructed Bland–Altman plots to assess the agreement of two standardized measures by studying the mean difference and constructing limits of agreement [40]. Correlation studies the relationship between one variable and another, not the differences, and it is not recommended as a method for assessing the comparability between methods. With this technique, the mean error score and the 95% prediction intervals can be examined in graphical form. The measures that are in closer agreement will have a mean bias close to zero and tighter 95% prediction intervals. We conducted all statistical analyses with R, version 3.2.3. 

## 3. Results

### 3.1. Demographic Data

Among the 20 eligible participants, 16 of them (80%) completed the three-month study. Four of the participants did not complete the study; two could not be contacted, and two dropped out because they found the trackers uncomfortable to wear. Characteristics of the participants are illustrated in Table 1. The rationale for recruiting active participants is that older adults who have a regular exercise regime without prior experience of activity tracker use would be more motivated to adopt activity trackers while being attuned to any shortcomings and challenges related to device usage. Additionally, some data points are missing due to a lack of device synchronization, device malfunctioning, and so on. As a result, 7.8% of the overall data points have been discarded for these reasons.

We evaluated participants’ physical activity readiness using PPAQ, a short, self-reported questionnaire that measures participation in leisure-time physical activity [41]. On average, participants reported their health as good or very good, and they engaged in various PA at a normal rate (2 to 3 mph) daily. Overall, 12 participants indicated that they regularly engaged in vigorous activities such as digging in the garden, strenuous sports, sustained swimming, brisk walking, and bicycling on hills. The remaining participants were involved in frequent light activities such as office work, driving a car, strolling, and personal care. All participants enjoyed seasonal sports and recreational events other than regular exercise classes at the fitness center.

### 3.2. Activity Tracker Adoption Attitudes Survey

Table 2 presents the tracker adoption survey results. Ten constructs were categorized into PA data privacy functions (health information sensitivity, legislative protection, perceived privacy risk) and benefits of using an activity tracker (personal innovativeness, perceived informativeness, functional congruence, perceived benefit, perceived prestige, adoption intention, and actual adoption behavior). The survey results indicated that participants were less concerned about the privacy risk of PA data (*M* = 3.78, *SD* = 1.10), recognized perceived functions and benefits well (*M* = 3.78, *SD* = 1.10), and showed positive adoption intention and behaviors (*M* = 3.78, *SD* = 1.10). Most participants were aware of health information sensitivity and did not consider PA data from the activity trackers as sensitive personal health information. Thus, they were comfortable with disclosing simple PA data to an external agent. Particularly, the appropriate legislative protection of their health data was considered as a facilitator for the adoption of wearable health monitoring devices (*M* = 5.29, *SD* = 1.43). Participants understood the benefits and values of activity tracker use well, and they showed positive attitudes toward device adoption (*M* = 3.78, *SD* = 1.10).

The non-parametric Friedman test and post-hoc Dunn’s test of differences among repeated measures of overall survey responses showed that temporal patterns were mixed, and significant periodic differences existed (*χ*^2^(2, 48) = 5.77, *p* = 0.0559). Specifically, participants’ concerns about the privacy of their activity data (health information sensitivity, legislative protection, and perceived privacy risk) were not notably decreased after they began using the activity tracker (*χ*^2^(2, 48) = 5.51, *p* = 0.064; *χ*^2^(2, 48) = 5.48, *p* = 0.0065; *χ*^2^(2, 48) = 5.21, *p = 0.074*, respectively). However, the responses regarding personal innovativeness, perceived informativeness, and functional congruence showed a decreasing pattern (*χ*^2^(2, 48) = 6.12, *p* < 0.05; *χ*^2^(2, 48) = 6.20, *p* < 0.05; *χ*^2^(2, 48) = 7.34, *p* < 0.05, respectively). The initial positive response about adoption intention and actual adoption behavior was not maintained throughout the entire study period (*χ*^2^(2, 48) = 7.67, *p* < 0.05; *χ*^2^(2, 48) = 6.19, *p* < 0.05, respectively). 

### 3.3. PA Performance Metrics 

**Figure 1:**
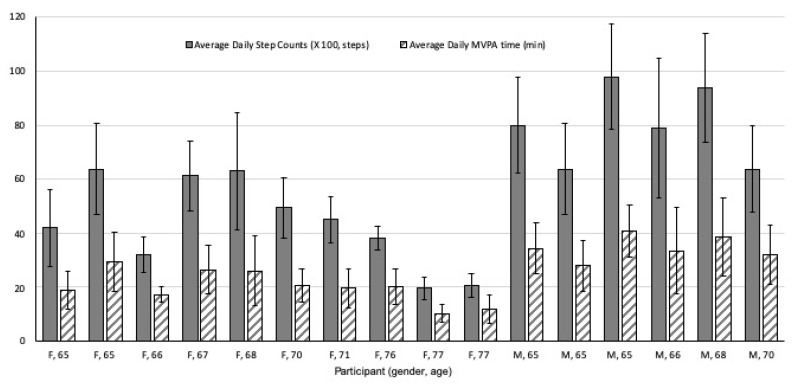
Averages of daily step counts and moderate and vigorous physical activity (MVPA) times for each participant.

Each participant’s step count and MVPA time data are presented in Figure 1. The mean number of daily steps averaged over all participants is 5,705 (*SD* = 1770), and the average MVPA time is 25.46 (*SD* = 10.35) min per day. This amount is higher than the average daily steps for their age groups suggested by the National Health and Nutrition Examination Survey (average 3733 steps). The average MVPA time is more than 150 min per week, which is the recommended amount for healthy adults [30,31].

**Figure 2:**
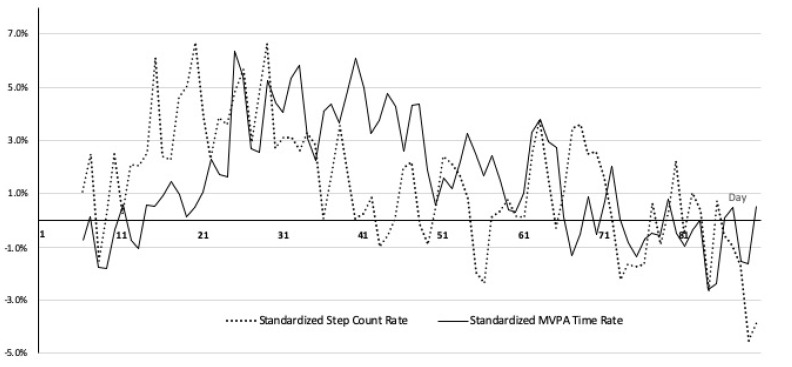
Standardized step count rate and Standardized MVPA time rate.

Figure 2 illustrates two PA measures: the standardized change rates of daily step counts and MVPA times by seven-day moving averages for all participants. We used a seven-day moving average because normally people’s PA has a weekly cycle. We conducted a one-way, repeated measures ANOVA to compare the effect of activity tracker adoption on these standardized change rates. The results showed that the activity tracker adoption elicited statistically significant differences in both measures over the course of the study (*F*(83, 1357) = 12.56, *p* < 0.00001: standardized step count rates, *F*(83, 1357) = 13.94, *p* < 0.00001: standardized MVPA time change rates), which shows that both MVPA time and step counts were significantly varied in the study period and the patterns followed similar waning patterns to survey outcomes.

**Figure 3:**
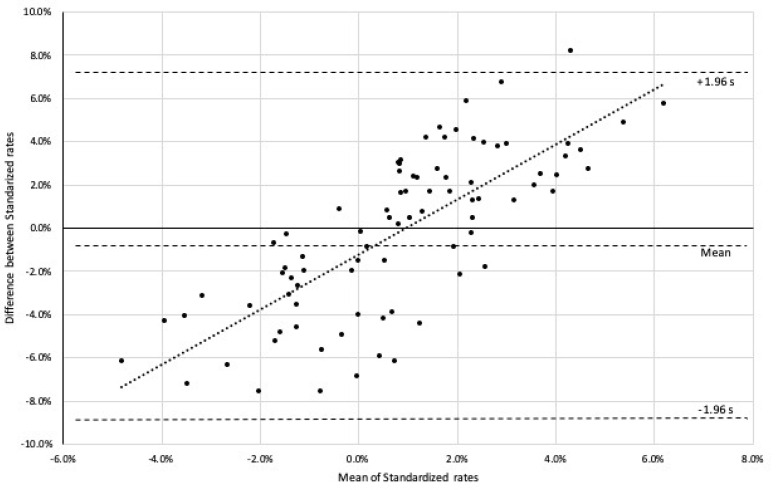
Bland–Altman diagram for deference between standardized rates (mean = −0.16%, standard deviation (s) = 3.76%) Dotted line represents the regression line (y = 1.2714x − 0.0119).

We conducted the Pearson correlation analysis in order to identify the relationship between the two standardized rates. The analysis did not provide the evident correlation between the two measures (*r* = 0.268, *p* = 0.284). As a further assessment of the agreement and comparability between two measures, we drew a Bland–Altman plot for the statistical behaviors of the difference between standardized rates and means of standardized rates (see Figure 3). The Bland–Altman plot showed agreement between two standardized rates by constructing limits of agreement [40]. The plot showed the bias and a range of agreement, within which were 95% of the differences between the two rates. The bias is not significant, however, because the line of equality is within the confidence interval of the mean difference, which could define whether the agreement interval was sufficiently narrow. Figure 3 illustrates the bias and range of agreement, within which were found 95% of the differences between one measurement and the other. It indicated that the bias is significant, because the line of equality is not within the confidence interval of the mean difference. 

## 4. Discussion

### 4.1. Waning Patterns of Adoption Attitudes and PA Performance

In this study, we examined active seniors’ subjective attitudes toward the adoption of the activity tracker, and PA performance (daily step counts and MVPA times) to understand the long-term effects of device adoption. Given that the participants were novice users of activity trackers, we assumed that the continuous use of the activity trackers might increase the participants’ step counts and MVPA times. Although some cyclical patterns of PA performance were observed, initial increases in PA performance could not be maintained across the study period. The survey results also confirmed these decreasing patterns. The participants showed positive initial attitudes on activity tracker adoption, and they were willing to share their PA records with others without reluctance. However, these positive adoption attitudes were not maintained, except for privacy-related constructs. These patterns indicate that the participants lost motivation and interest in the active use of the devices over a protracted period and disregarded the benefits of long-term use. 

The waning patterns are more evident in self-efficacy items in the survey responses. Among various measured attitudes, self-efficacy has been known to be the most consistent predictor of both the adoption of activity trackers and PA maintenance [21,42]. The waning patterns in self-efficacy items mean that simply providing an activity tracker would not be enough to make participants more active. This finding strengthens the importance of having a tailored self-efficacy intervention, such as offering individualized goal setting that enables a gradual increase in activity levels or developing self-regulatory skills to meet minimum daily exercise time goals [43,44].

Another major cause of the waning patterns is the novelty effect. The novelty effect has been defined as a person’s subjective response to technology usage, not the pattern of usage that will persist over time as the product ceases to be new to initial users [45]. A prior study noted that, as the novelty effect diminishes, many users stop using new technologies [46]. The survey results identified participants’ positive attitudes and their intentions related to the activity trackers at the beginning of usage, but this confidence waned as they became familiar with their devices. Furthermore, the novelty of activity trackers may negatively influence adoption behaviors because the participants are often reluctant to change routine behaviors. In addition, novelty may not be enough of an incentive to increase step counts. When using a new technology requires the implementation of radical changes in current behaviors, its adoption can be negatively affected. The underlying causes for these issues were that the new devices did not meet users’ expectations, the information provided by the devices was deemed to not be beneficial, and the maintenance required effort [46].

### 4.2. Different Onset and Duration of PA Performance Metrics

Interestingly, although the Bland–Altman plot confirmed both measures are valid for assessing PA levels, they showed distinctive cyclic patterns. The correlation between the two parameters is low, and there is a clear trend in the BA plot, meaning they are not interchangeable. Step counts increased in earlier periods, and MVPA times arose and lasted longer in later periods. The ANOVA and the Pearson correlation result also verified that step counts and MVPA times showed different patterns (i.e., onset timing and increased durations) over a longer duration of activity tracker adoption. Earlier onset of step count increases implied that participants are more likely to increase their step count when motivated by the adopted activity tracker. Overall, step counts measure overall PA performance, but MVPA time provides more exercise-oriented PA measurements. 

Generally, step count has been used as a major indicator of PA levels [47,48,49]. Achieving a daily step count goal is recommended not only for positively influencing individual physical health, but also for improving self-efficacy and cognitive well-being [47]. However, the accuracy of activity trackers is disputable, and several studies have reported the validity of assessing step counts [50,51]. Some studies have found a strong reliability of step counting in activity trackers [52], but others have questioned their accuracy, even in ambulatory settings [53]. When comparing MVPA times, it is not apparent whether daily step counts would have a positive motivational effect over a long-term period. Given that a majority of activity tracker studies are short term [49], we can expect a temporary PA increase due to the novelty of wearing an activity tracker and the participants’ striving to achieve step count goals.

These distinctive patterns of two parameters demonstrate the unique features of older adults’ activity tracker adoption. Considering older adults’ slow walking/running speed, irregular stride, and more sedentary time, step counts would not be an appropriate indicator of activity tracker adoption. Due to inaccurate measurements of stride length and step counts, MVPA based on energy expenditure may lead to an appropriate exercise and PA recommendation [54]. Midorikawa et al. [55] suggested that relationships between PA intensity and step counts are different between locomotion and the other types of PA, and step counts do not assess various types of PA other than locomotion. While the step counts show the limited activity data focusing on walking, running and some other cardiovascular exercises, PA intensity based on activity periods may reflect more effective patterns of activity tracker use, particularly for older adults. Health benefits of older adults depends not just on the number of steps but on total energy expenditure due to physical activity. In addition, MVPA times make it easier to track the recommended guidelines, which is shown as the time of moderate-intensity aerobic activity or vigorous-intensity aerobic activity. 

### 4.3. Strengths and Limitations of the Study

This study has several strengths. The longitudinal design furnished data at different time points, thereby providing more analytic windows than a cross-sectional design would. Because the participants routinely exercised, they were likely to be motivated and appreciated the potential benefits of using an activity tracker. These characteristics probably positioned them to identify explicit adoption patterns and other issues related to activity tracker usage. The participants had no prior experience using activity-tracking devices. Presumably, they had no biases regarding activity tracker adoption. Our results suggested that a relationship exists between subjective adoption attitudes and PA measures, including daily step counts and MVPA times over a long-term period. These two measures provide meaningful insights of actual tracker adoption outcomes in PA amounts and levels.

The limitations of this study include its small sample size and group homogeneity (i.e., active adults). These may preclude the findings from being generalizable to the larger population of older adults. Therefore, the results should be considered exploratory and interpreted with caution because they are intended to evaluate feasibility, rather than to formally test hypotheses or provide generalizable knowledge. These issues may also affect the reliability of the information from the survey instruments. The sample size considered for evaluation is limited, and future studies should consider performing a complete evaluation on a larger group of participants. 

Additionally, skepticism regarding the accuracy of activity trackers and their ability to capture various physical activities was another major barrier to adoption and activity tracker use. Activity trackers cannot capture all types of activities including bicycling and swimming due to the nature of the accelerometers used in the devices for tracking activity. Commercially available devices significantly overestimate or underestimate total activity [56]. Measures of total daily energy expenditure and MVPA were less accurate. Much variability was reported among the measurement accuracies across the devices [57]. 

The design and functions of the activity tracker used in this study are not optimized for older adults’ use. Though participants demonstrated high-level adherence over daily long-term use, there are possible errors in converting sensor signals to quantify step counts or to record the onset and completion time of activities. Activity trackers need to have acceptable accuracy, especially for measuring step counts, MVPA, and heart rates [58]. In addition, older adult-oriented designs, functional features, and usefulness are required to motivate older adults to use activity trackers to help them increase PA [59,60]. As a viable approach, Magistro et al. [61] suggested a step detection algorithm that is specifically designed to be sensitive to slow-speed ambulation, which is typical of older adults. An activity tracker that is tailored to older adults’ demands and capability is necessary to further explore the usability, feasibility, and validity of such devices. Finally, activity tracker experiments would be more appropriate in a laboratory setting than in a natural observational setting, which may include limited structured protocols regarding the evaluation and comparison of PA. Nevertheless, free-living conditions with unstructured scenarios can provide valuable insights into the adoption of activity trackers.

## 5. Conclusions

This study has made the following primary contributions: (a) we contrasted active seniors’ activity tracker adoption attitudes with actual PA measures to understand how subjective adoption attitudes are reflected in older adults’ usage of activity trackers, and (b) we uncovered previously underreported longitudinal changes regarding the adoption effect in older adults’ PA levels. 

In this study, we attempted to verify the adoption patterns implied from the subjective, construct-based survey and to compare them to measures of actual PA performance from activity tracker use. The results contributed to an evaluation of activity tracker adoption in a longitudinal way. Prior research has shown that the adoption of an activity tracker encourages both continuous and accumulated increases in PA and is associated with health benefits. However, it is unknown whether subjective positive attitudes are reflected in the improvement of PA performance or how participants’ performances can vary when highlighting the value of an accumulated period of long-term activity tracker adoption. Our results confirmed that similar adoption patterns were shown in both subjective adoption attitudes and actual PA measures. The results support the development of better activity tracker designs and motivational intervention for long-term use.

## Figures and Tables

**Table 1:** Demographic characteristics of participants (*N* = 16).

	Male	Female
Age (years)	67.5 ± 2.5	71 ± 6
Height (cm)	175.8 ± 6.7	164.6 ± 5.9
Weight (kg)	81.7 ± 5.77	69.1 ± 5.45
BMI (kg/m^2^)	26.47 ± 3.78	25.34 ± 3.79
Race		
Caucasian/White	2	4
Hispanic	4	5
Black	-	1

**Table 2:** Integrated Activity Tracker Adoption Survey Scale Results (mean (SD).

Constructs	Survey Items	Beginning (1st Week)	Middle (7th Week)	Ending (13rd Week)
Health information sensitivity	I feel comfortable with the type of health information the activity tracker request from me.	6.5 (1.43)	6.1 (1.29))	6.2 (1.52)
	I do not feel the activity tracker gathers highly personal health information about me.	6.1 (1.79)	5.9 (1.95)	6.0 (1.66)
	The health information I should provide to the activity tracker is not sensitive to me.	5.9 (1.49	5.7 (1.36)	5.8 (1.55)
Legislative protection	I believe that I would be protected from the misuse of my physical activity.	5.3 (1.89)	5.7 (1.36)	5.6 (1.67)
	I believe that the practices of how activity trackers collect, use, and protect my private health information should be governed and interpreted.	5.7 (1.55)	5.5 (1.78)	5.4 (1.88)
	I believe that the violation of the health information I provided to activity trackers should be able to be addressed.	4.5 (1.33)	4.4 (1.40)	4.5 (1.39)
Perceived privacy risk	It would be assured to disclose my physical activity information to activity tracker vendors.	4.6 (1.75)	4.7 (1.56)	4.5 (1.65)
	There would be low potential for loss associated with disclosing my physical activity information to activity tracker vendors.	3.9 (1.28)	3.7 (1.58)	3.7 (1.62)
	There would not be much uncertainty associated with giving my physical activity information to activity tracker vendors.	4.3 (1.44)	4.4 (1.51)	4.5 (1.29)
Personal innovativeness	If I heard about a new technology, I would look for ways to experiment with it.	4.9 (1.62)	4.7 (1.54)	4.7 (1.77)
	Among my peers, I am usually the first to try out new technologies.	4.9 (1.62)	3.8 (1.51)	3.9 (1.66)
	In general, I like to experiment with new technologies.	3.7 (1.71)	3.8 (1.44)	3.6 (1.52)
Perceived informativeness	Activity trackers are good sources of personal health information.	5.8 (1.89)	4.5 (1.21)	3.9 (1.37)
	Activity trackers supply relevant health information.	5.9 (1.78	4.3 (1.32)	4.0 (1.41)
	Activity trackers are informative about my personal health information.	5.8 (1.73)	4.3 (1.29)	3.5 (1.38)
Functional congruence	Activity trackers are (expected to be) comfortable.	5.0 (1.69)	3.7 (0.98)	3.4 (0.75)
	Activity trackers are (expected to be) durable.	5.8 (1.47)	5.0 (1.35)	4.5(1.33)
	Activity trackers are (expected to be) priced appropriately considering their quality.	5.2 (1.52)	5.0 (1.60)	4.6 (1.48)
Perceived benefit	Using an activity tracker would improve my access to my health information.	5.6 (1.31)	4.8 (1.33)	4.7 (1.24)
	Using an activity tracker would improve my ability to manage my health.	5.7 (1.49)	5.1 (1.39)	4.8 (1.47)
	Using an activity tracker would improve the quality of my healthcare.	4.8 (1.29)	4.2 (1.30)	4.0 (1.37)
Adoption intention(Self-efficacy)	I will be able to achieve most of the health goals that I have set.	5.5 (1.77)	3.0 (1.04)	2.4 (0.85)
	I can obtain desirable health outcomes that are important to me by activity tracker use.	4.9 (1.48)	3.1 (1.25)	2.7 (0.89)
	I am confident that I can exercise effectively with an activity tracker.	5.1 (1.25)	3.2 (1.07)	2.7 (0.77)
Actual adoption behavior	I use an activity tracker to stay on the path of healthy living.	5.3 (1.30)	4.2 (1.13)	3.4 (0.91)
	I often use an activity tracker to get health information.	4.7 (1.33)	3.2 (0.96)	2.6 (0.76)

7 Likert scale was used (7: Strongly agree, 6: Agree, 5: Somewhat agree, 4: Neutral, 3: Somewhat disagree 2: Disagree, 1: Strongly disagree).
